# Study of Transfer Characteristics of a Molecular Electronic Sensor for Borehole Surveys at High Temperatures and Pressures

**DOI:** 10.3390/s19112545

**Published:** 2019-06-04

**Authors:** Ilya Evseev, Dmitry Zaitsev, Vadim Agafonov

**Affiliations:** Department of Physical and Quantum Electronics, Moscow Institute of Physics and Technology, 141701 Moscow, Russia; zaitcev.dl@mipt.ru (D.Z.); agafonov.vm@mipt.ru (V.A.)

**Keywords:** molecular electronic technology (MET), high temperature sensor, extended temperature range, transfer function, borehole, oil production, gas production, profiling

## Abstract

The paper considers the development and experimental study of the characteristics of a high-temperature motion parameter sensor based on molecular-electronic technology (MET) operating at elevated pressures. Studies were conducted in an extended temperature range (25–125 °C) with a static external pressure of up to 10 atm. A pilot plant based on a high-pressure chamber with the ability to output an electrical signal was specially designed and commissioned. A family of amplitude-frequency characteristics of a ME sensor in an extended temperature range was obtained for the first time. A theoretical model was constructed and verified to describe the transfer function of the sensor at high temperatures and pressures. The activation energies of active carriers were calculated, and a prediction was made about the possibility of using the developed devices for the needs of the oil and gas mining industries.

## 1. Introduction

Modern oil wells are longer, deeper, and hotter than ever before. This is related to the depletion of easily accessible hydrocarbon deposits and the need for deep-seated deposit development. As oil and gas production becomes more and more technically complex, operators must introduce innovative technologies and new methodologies to optimize the process of exploration and development of oil and gas fields [1]. There is a trend to increase the depth of oil wells. For example, in the Gulf of Mexico, in comparison with the 1990s, the maximum depth of the wells increased by 40% (from 7 to 10 km). Such depths are characterized by high temperatures (150–260 °C) and pressures (69–241 MPa) [2].

The design, maintenance, and operation of deep wells, as well as methods of sounding the interwell space, require the use of specific elemental bases. Classical equipment (accelerometers, inclinometers, geophones, and hydrophones) used to explore shallow deposits is not suitable for these purposes. Therefore, many companies develop their own measuring systems (for example, the Honeywell DHTA230 high-temperature well accelerometer [3]). There are not many technologies on the basis of which sensitive elements for such extreme conditions can be created. Some solutions based on fiber-optic sensors have been offered [3,4,5]. There are solutions based on MEMS technologies [6]. Fluxgate magnetometers and NMR sensors for drilling navigation are also used to solve certain problems [7]. Each technology has its advantages and disadvantages.

One of the main methods of wellbore space exploration is vertical seismic profiling (VSP) [8]. Generally speaking, its essence is that a seismic probe is put inside the well, where there are sensitive elements fixing elastic waves from a certain signal source (vibration source, explosions, etc.) on the surface. Further, the received signal is processed and a model near the well or interwell space is constructed [9,10].

In addition to stable operation at high temperatures and pressures, sensor elements for seismic probes should have sufficient sensitivity. One of the interesting solutions could be the use of sensors based on molecular-electronic technology (MET) for such extreme environmental conditions [11,12].

Molecular electronic sensors of motion parameters are extremely sensitive, and they are compact enough to fit in an intraprobe space. In comparison with classical geophones (electromagnetic), MET sensors have a number of significant advantages:

(1) Significantly higher (10 times) sensitivity (in particular at low frequencies. This is very important for seismic applications);
(2) Low noise at low frequencies.

Despite the use of liquid inertial mass, the combination of high temperature and high pressure significantly shifts the boiling point of the electrolyte used in the system, allowing the MET-based sensor to function successfully in such difficult environmental conditions. Besides, MET-based meters can be used both as classic geophysical sensors (geophones, accelerometers) [13,14] and as acoustic pressure sensors [15,16], which increases the variability and complexity of research, as well as increasing the variety of tasks for the solution of which they can be applied. In this context, MET-based meters could be considered as an alternative to the technologies already used for such studies, under the condition of the experimental and theoretical rationale of their proper functionality under extreme conditions of high temperatures and pressures.

In the framework of this study, a MET-based high-temperature motion parameter sensor was developed. To study the sensor behavior in high temperature conditions, a special thermal pressure chamber was developed and put into operation. The amplitude frequency response of the sensor in an extended temperature range with external static pressure was received. A theoretical model of the MET amplitude-frequency characteristic, which describes the characteristics of the frequency response at high temperatures and pressures, was developed and experimentally verified.

**Figure 1:**
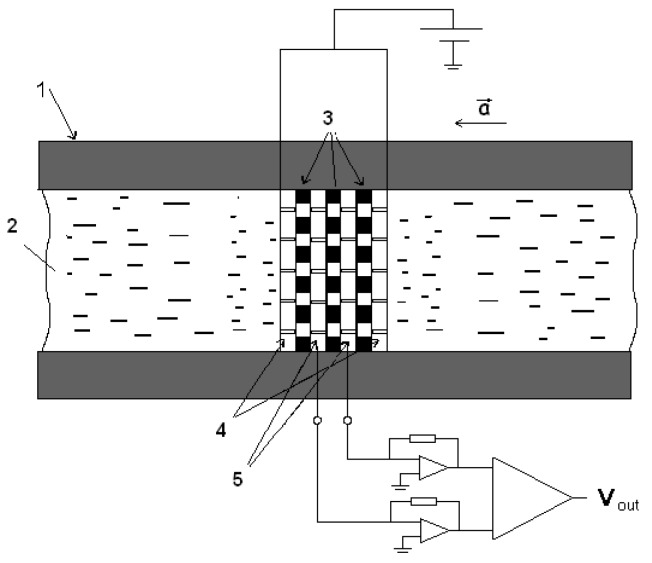
Schematic diagram of the molecular electronic transducer (MET). 1—converter housing, 2—working fluid, 3—dielectric spacers, 4—anodes, 5—cathodes (3-4-5—electrode node).

**Figure 2:**
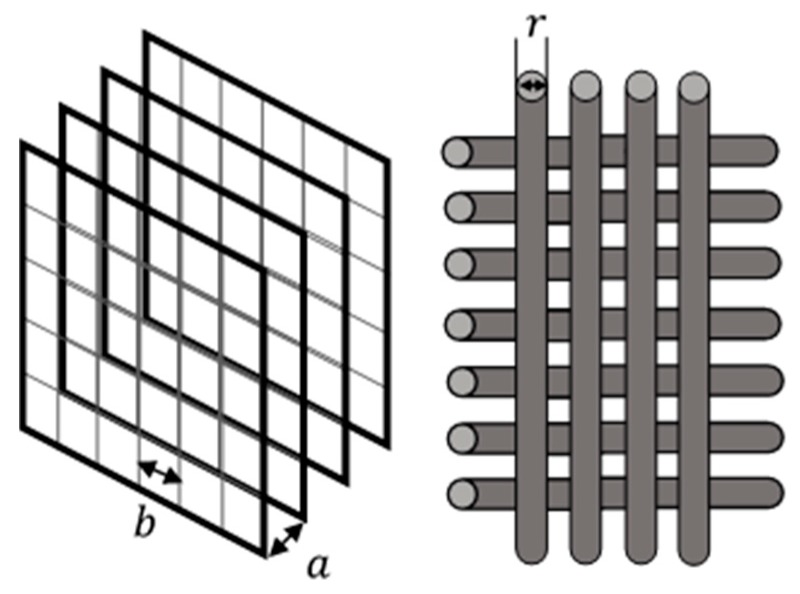
Diagram of the electrode assembly indicating the overall parameters. *a* is the distance between the electrodes, *b* is the distance between the threads of the electrode grid, *r* is the radius of the electrode thread).

Theoretical Part:

Any ME-based device consists of a MET conversion element, schematically shown in Figure 1: the device case (with or without elastic elements); the electrolyte, which is simultaneously the inertial mass for the accelerometers, geophones, and the medium transmitting variations of acoustic pressure for hydrophones; and an electronic board that specifies the operating point in voltage between the electrodes and providing signal currents from the cathodes. More information about the basic principles can be found in [17,18]. Figure 1 and Figure 2 show a schematic diagram of the device and the layout of the electrodes of the electrode assembly, respectively.

The device operation principle is as follows: Under the influence of external acceleration $\vec{a}$, there is a convective transfer of the main current carriers in the electrolyte, which are usually (*J_3_^−^*) ions, from the anodes to the cathodes, thereby changing the cathode current proportional to the external effect. The efficiency of such conversion depends, in the simplest terms, on the mechanical subsystem defined by hydrodynamic resistance, rigidity, and shape [19], as well as the electrochemical subsystem determining the efficiency of converting fluid flow into current and depending on the electrolyte composition, electrode design, and electrochemical conversion mechanisms [20]. Thus, the transfer function of the MET can be defined as follows

(1) $$W=\frac{J_{signal}}{a}$$

where  a is the acceleration of the object (for example, the Earth’s surface) and $J_{signal}$ is the output current of the cell caused by a mechanical signal. For a known concentration distribution, the currents through the electrodes can be found by this equation [20]:

(2) $$J_{signal}=-Dq\overset{}{\underset{S}{\oint }}\left(\nabla c,\vec{n}\right)dS$$

where integration is performed over the electrode surface S, $\vec{n}$ is a unit vector normal to the surface, q is the charge transferred through the electrode in a single reaction, *D* is the diffusion coefficient, and c is the concentration of the main current carriers.

Meanwhile, the total transfer function of the entire system is expressed as:

(3) $$W=W_{mech}*W_{el-ch}$$

where $W_{mech}$ and $W_{el-ch}$ are transfer functions of the mechanical and electrochemical systems, respectively.

Previous studies [21] have shown that the frequency response of a MET sensor can be quite accurately described by the expression:

(4) $$W\left(\omega \right)=\frac{\frac{A_{0}}{\omega }}{{\left(1+\frac{\omega_{mech,1}{}^{2}}{\omega^{2}}\right)}^{\frac{1}{2}}\;{\left(1+\frac{\omega_{mech,2}{}^{2}}{\omega^{2}}\right)}^{\frac{1}{2}}\;\;{\left(1+\frac{\omega^{2}}{\omega_{el-ch}^{2}}\right)}^{\frac{1}{2}}\;\;{\left(1+\frac{\omega^{2}}{\omega_{D}^{2}}\right)}^{\alpha }}$$

where $A_{0}$, $\omega_{mech,1}$, $\omega_{mech,1}$, $\omega_{el-ch}$, $\omega_{D}$, and α are the approximation parameters.

Here, $W_{mech}$ is defined by Equation (5), and $W_{el-ch}$ is defined by Equation (6).

(5) $$W_{mech}\left(\omega \right)=\frac{\frac{A_{0}}{\omega }}{{\left(1+\frac{\omega_{mech,1}{}^{2}}{\omega^{2}}\right)}^{\frac{1}{2}}\;{\left(1+\frac{\omega_{mech,2}{}^{2}}{\omega^{2}}\right)}^{\frac{1}{2}}\;}$$

(6) $$W_{el-ch}\left(\omega \right)=\frac{\frac{A_{0}}{\omega }}{\;\;{\left(1+\frac{\omega^{2}}{\omega_{el-ch}^{2}}\right)}^{\frac{1}{2}}\;\;{\left(1+\frac{\omega^{2}}{\omega_{D}^{2}}\right)}^{\alpha }}$$

$\omega_{el-ch}$ and $\omega_{D}$ are defined by Equations (7) and (8), respectively.

(7) $$\omega_{el-ch}\sim \frac{D}{a^{2}}$$

(8) $$\omega_{D}\sim \frac{D}{r^{2}}$$

This parameter shows how long a charged triiodide ion (*J_3_^−^*) will diffuse through distances a and *r* (Figure 2).

The key parameters of the MET subject to serious changes with increasing temperature are the diffusion coefficient D and the viscosity coefficient η for the electrolyte [22].

The diffusion coefficient D is determined by the ratio from [22] as:

(9) $$D=\frac{\delta^{2}}{2\tau }\exp\left(-\frac{U}{kT}\right)$$

where k is the Boltzmann constant, T is the temperature, r is the molecule radius, η is the viscosity coefficient, τ is the time of a single particle oscillation in a potential well, δ is the distance traveled by a particle during an elementary act, and U is the activation energy.

As it can be seen from Equation (9), the diffusion coefficient D strongly depends on the temperature T.

## 2. Experimental Part

### 2.1. Experimental Setup

**Figure 3:**
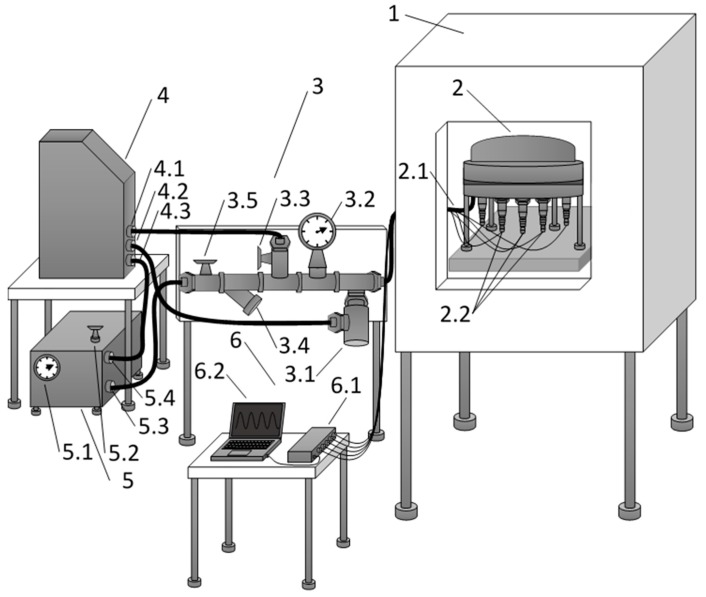
Thermal vacuum chamber design. 1—Heat chamber (M-60/100-120 KTX-T); 2—Pressure chamber, 2.1—High pressure supply hose, 2.2—Electrical signal outputs from the sensors; 3—Hydraulic system control unit, 3.1—safety valve, 3.2—barometer, 3.3—pressure relief valve, 3.4—filter, 3.5—shut-off valve; 4—Container with working fluid, 4.1—hose for draining the working fluid, 4.2—hose for draining the relief valve, 4.3—hose for supplying the working fluid; 5—Crimping tool, 5.1—barometer, 5.2—pressure adjustment valve, 5.3—output high-pressure connection, 5.4—input connection of the working fluid supply; 6—Signal processing unit, 6.1—ADC (Analog-Digital Converter), 6.2—PC (Personal Computer).

To perform the research tasks on the study of the MET functioning under the conditions of high temperatures and pressures, a special experimental setup (thermal chamber) was designed and put into operation. Its design is shown in Figure 3.

**Figure 4:**
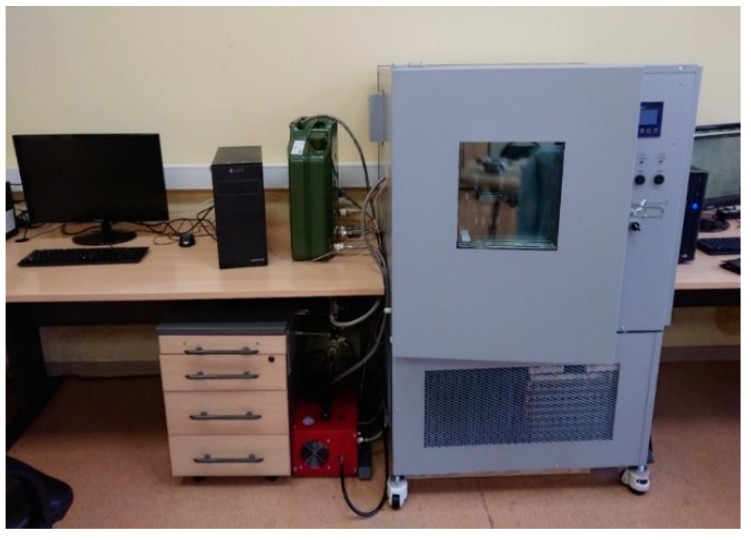
General appearance of the installation.

**Figure 5:**
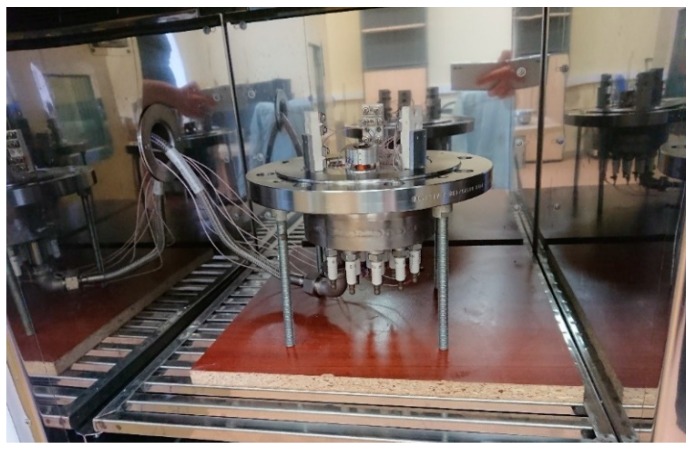
Clear compression chamber.

The installation appearance is presented in Figure 4 and Figure 5

**Figure 6:**
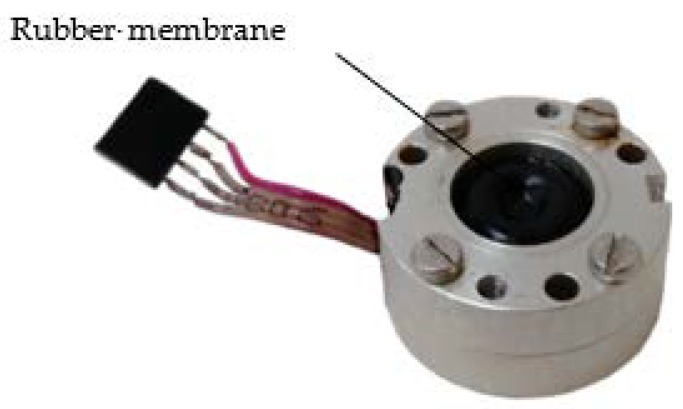
Molecular-electronic technology (MET)-based accelerometer.

**Figure 7:**
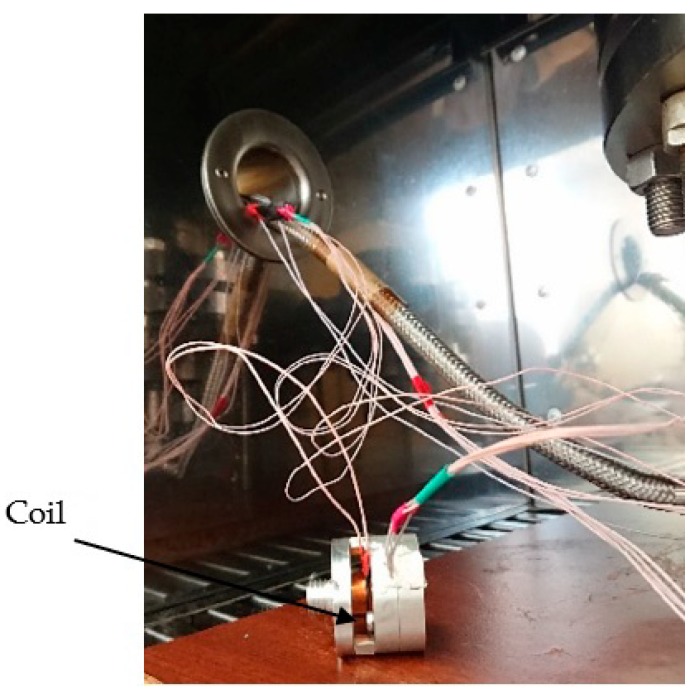
Accelerometer with conversion electronics and feedback circuit.

Figure 6 and Figure 7 show the developed high-temperature MET sensor. Figure 6 shows the sensitive element (accelerometer), while Figure 7 shows a sensing element assembly to study the amplitude–frequency characteristics.

Excessive pressure in the chamber is created with the help of a crimping tool (5). The working fluid (silicone PMS-50) is in a closed container. Pipe fittings are connected to it to drain and supply the working fluid (4.1, 4.2, 4.3). The liquid is supplied to the inlet piece (5.4). Then it is fed under pressure to the input of the hydraulic control unit (3).

The intensity of crimping is regulated by a valve (5.2). The pressure in the system is monitored with the help of a barometer (3.2). The safety valve (3.1) releases the working fluid from the system when the specified pressure is exceeded. The pressure can be manually released with a valve (3.3). In order to prevent large particles from entering the system, a filter (3.4) was installed. Isolation of the crimping tool from the rest of the system is carried out using a shut-off valve (3.5).

The studied sensors are securely fixed inside the pressure chamber (2) and are connected to the electrical outputs of the signal from the sensors (2.1). The electrical leads are connected to the ADC (Analog-Digital Converter) (6.1) located outside the high pressure and temperature zones.

The study of the transfer function of the sensor was carried out by the method described in detail in [23]. A magnet was attached to one of the membranes, an electromagnetic coil was located on the outer mount so that the magnet could easily move inside it. The known sinusoidal signal was fed from the output of the ADC (6.1) to the coil. Thus, the variable electromagnetic field arose into the coil, so the magnet with the membrane was pulled into the coil. The membranes created a flow of electrolytes through the transducer. Then, the converted electrical signal went to the differential input of the ADC (6.1). Then, the received analog signal was converted to digital and fed in real time at PC (Personal Computer) (6.2). Thus, the sensor response and the calibration signal were recorded at ~40 frequencies from a range of 0.1–480 Hz. Then, the ratio of the amplitudes of the spectral responses of MET and the reference signal was calculated. After the data processing, the transfer function could be built.

A high-temperature MET-based accelerometer has a number of important differences compared to the classical MET sensor [23]. One difference is that there are no polycarbonate elements since at temperatures of 130–150 °C it softens to a state of clay. The body is made of aluminum alloy. Membranes for the sensor are made of high-temperature rubber, for the same reason. Moreover, the calibration circuit involves extra high (EH) class magnets, since ordinary N class magnets (Normal) are demagnetized at temperatures of about 80 °C.

### 2.2. Experimental Data Processing

**Figure 8:**
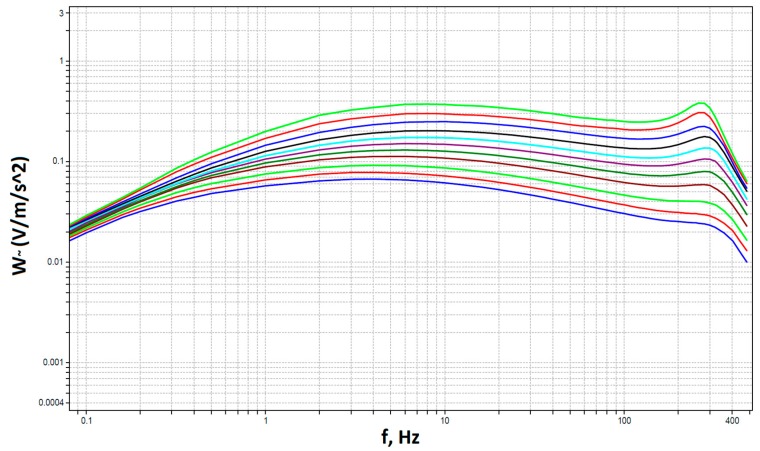
Transfer characteristics of a molecular electronic sensor in an extended temperature range (25–125 °C) with an external static pressure of 10 atm. The Y axis shows the sensor response W (~V/m/s^2^), the X axis shows frequency f (Hz). Logarithmic scale. The obtained curves were further approximated using Equation (4). Figure 9 and Figure 10 show the sensor transfer functions and the approximation curves at certain temperatures.

This research was the first one to help to obtain experimentally the transfer characteristics of a MET sensor in an extended temperature range (25–125 °C) with an external static pressure of 10 atm. The obtained characteristics are given in Figure 8. Based on the graph, there was a significant increase in the sensor sensitivity with increasing temperature, which coincides with the results of previous studies [24].

It is worth noting that in real seismic applications external pressure may be bigger than 10 atm. During the study, a series of experiments were conducted to explore the dependence of the transfer function of the MET sensor on pressure in the pressure range (1–10 atm). As a result, there was no difference between the form of the curves at different pressures. Furthermore, the survival of MET sensors at high pressures (100 atm.) was checked. The sensors stayed in work condition.

**Figure 9:**
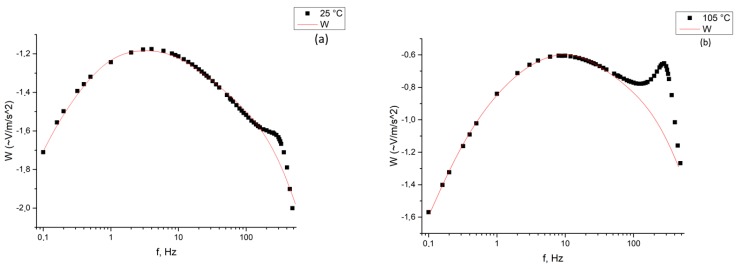
The original (black) and approximated (red) characteristics of a molecular electronic converter at the temperatures of 25 and 105 °C, respectively, and the pressure of 10 atm. The Y axis shows the response of the sensor W (~V/m/s^2^), the X axis shows the frequency f (Hz): (**a**) characteristics of a molecular electronic converter at the temperature of 25 °C; (**b**) characteristics of a molecular electronic converter at the temperature of 105 °C.

**Figure 10:**
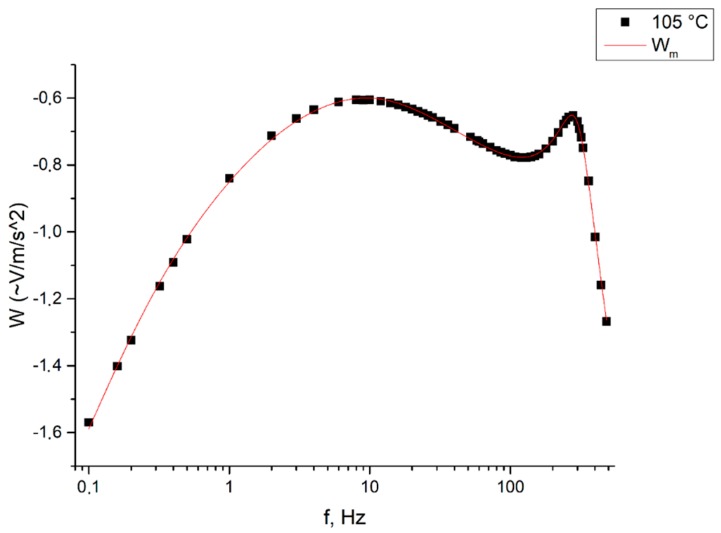
Initial (black) and approximated (red) characteristics of a molecular electronic converter at a temperature of 105 °C and a pressure of 10 atm. The Y axis shows the response of the sensor W (~V/m/s^2^), the X axis shows the frequency f (Hz).

The obtained curves were further approximated using Equation (4). Figure 9 and Figure 10 show the sensor transfer functions and the approximation curves at certain temperatures.

As it can be seen from Figure 9, already at 25 °C and elevated pressure, Equation (4) generally describes the characteristic at low frequencies quite well, but, unfortunately, does not describe the typical peak of the experimental dependence arising at the frequency of ~300 Hz. This is especially noticeable at high temperatures (for example, 105 °C), where a typical shift of the peak towards lower frequencies is seen.

In this regard, it was decided to abandon the simplified model from Equation (4) and to formulate a more general concept that considers the resonant nature of the mechanical subsystem and the presence of at least three typical dimensions of electrode assembly, Figure 2, which can affect the electrochemical subsystem. As a result, the proposed analytical dependence of the transfer function of the MET accelerometer was formulated for high temperature and pressure conditions in Equation (10):

(10) $$W_{}\left(\omega \right)=\frac{\frac{A_{0}}{\omega }}{{\left({\left(1-{\left(\frac{\omega_{0}}{\omega }\right)}^{2}\right)}^{2}+\frac{d^{2}\omega_{0}^{2}}{\omega^{2}}\right)}^{\frac{1}{2}}\;{\left(1+\frac{\omega^{2}}{\omega_{D_{1}}^{2}}\right)}^{\frac{1}{4}}\;\;{\left(1+\frac{\omega^{2}}{\omega_{D_{2}}^{2}}\right)}^{\frac{1}{4}}\;\;{\left(1+\frac{\omega^{2}}{\omega_{D_{3}}^{2}}\right)}^{\frac{1}{4}}}$$

Here, the mechanical part of $W_{mech}$ is replaced by the general formula for an oscillatory system with a natural frequency $\omega_{0}$ and a damping coefficient d, similar to the one presented in [25]. The electrochemical part of $W_{el-ch}$ has also been presented more generally. Now it takes into account three possible physical mechanisms of influence of the overall parameters of the electrode node on the electrochemical transfer function of the system.

In addition to the frequencies $\omega_{el-ch}$ and $\omega_{D}$ (in new notations $\omega_{D_{1}}$ and $\omega_{D_{3}}$), a new diffusion frequency $\omega_{D_{2}}$ related to the distance between the threads of the electrode grid was introduced by Equation (11):

(11) $$\omega_{D_{2}}\sim \frac{D}{b^{2}}$$

We tried to approximate the dependencies found experimentally using the modified analytical Equation (11). Figure 10 shows the sensor transfer function at 105 °C, approximated by Equation (11). As it can be seen from Figure 10, Equation (10) describes the obtained experimental characteristic with good accuracy.

For each of the transfer functions, taken at different temperatures, the corresponding approximation parameters $\omega_{0}$, d, $\omega_{D_{1}}$,$\;\omega_{D_{2}}$, $\omega_{D_{3}}$ were selected.

Table 1 below presents these approximation parameters for each temperature.

Based on Table 1, the parameter $A_{0}$ (physically, it is the sensor response at the lowest frequencies) in the studied temperature range can be considered constant, which correlates well with the experimental data ($A_{0}$ weak dependence on temperature (Figure 8)). The diffusion frequencies $\omega_{D_{1}}$,$\;\omega_{D_{2}}$, and $\omega_{D_{3}}$ increased with increasing temperature, which correlates well with the theoretical assumption about their diffusion nature. It happens because of the exponential growth of the diffusion coefficient (9). The typical shift of the natural frequency $\omega_{0}$ from 360 Hz at 25 °C to 298 Hz at 125 °C was observed. This was due to the softening of the rubber membranes, which led to a decrease in the rigidity of the mechanical system, which, in turn, affected the natural frequency $\left(\omega_{0}\right)$. Along with this, there was an increase in the sensor response at the resonant frequency of the mechanical system $\left(\omega_{0}\right)$. This effect is associated with a decrease in the viscosity of the electrolyte with increasing temperature, which led to a decrease in the damping of the mechanical system (d).

For this research, only one experimental sample of MET sensors was used, so a statistical analysis of the observed patterns was not carried out.

## 3. Verification of Found Patterns

Thus, the temperature dependence of the parameters of the transfer function of the sensor was experimentally obtained. Based on Equation (9), the diffusion coefficient depends on temperature exponentially. Let us try to approximate the dependences $\ln\left(\omega_{D_{1}}\right)\left(\frac{1}{T}\right)$, $\ln\left(\omega_{D_{2}}\right)\left(\frac{1}{T}\right)$, and $\ln(\omega_{D_{3}})\left(\frac{1}{T}\right)$ by a linear function using the method of least squares and to find the angular coefficient of the obtained straight line, which, based on the theory of Frenkel [21], has the meaning of the activation energy of triiodide ions in the electrolyte.

**Figure 11:**
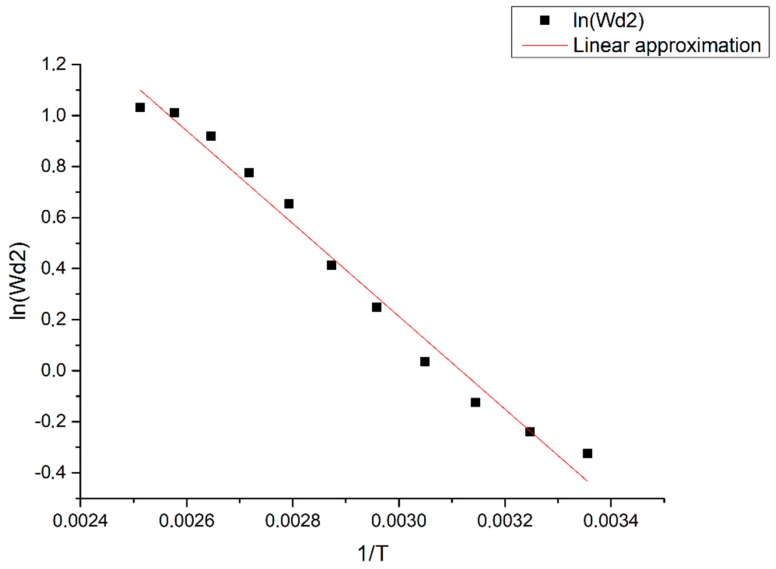
Approximation of the slope coefficient for $\ln\left(\omega_{D_{2}}\right)$. The X axis shows the inverse temperature (1/K), while the Y axis shows the approximated values of $\ln(\omega_{D_{2}})$.

Figure 11 shows an example of the dependence $\ln\left(\omega_{D_{2}}\right)\left(\frac{1}{T}\right)$ (for $\ln\left(\omega_{D_{1}}\right)\left(\frac{1}{T}\right)$ and $\ln(\omega_{D_{3}})\left(\frac{1}{T}\right)$ it is constructed in a similar way). The results of the calculation of the angular coefficients are presented in Table 2.

In accordance with Equation (2), the signal background current (in the absence of strong perturbations) is also proportional to the diffusion coefficient of the triiodite ions. We checked if the values found for the activation energies coincided (using the found approximation parameters $\omega_{D_{1}}$,$\;\omega_{D_{2}}$, $\omega_{D_{3}}$) in accordance with Figure 11 with an alternative calculation of the diffusion coefficient dependence associated with the background current.

**Figure 12:**
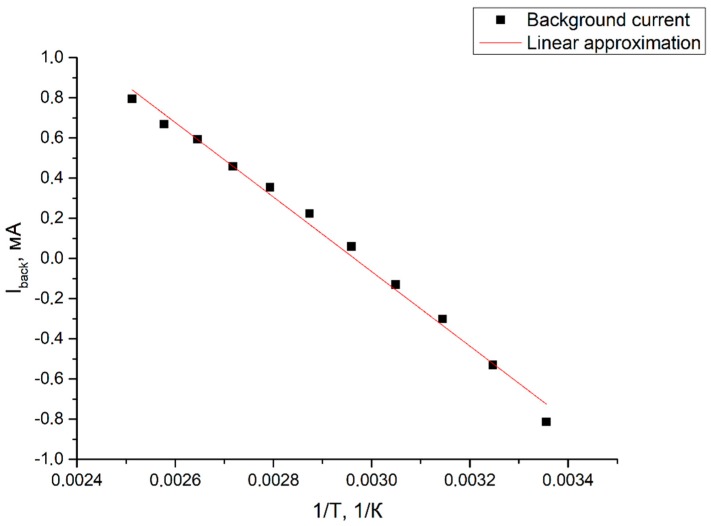
Background current *I*_background_ dependence on inverse temperature (1/*T*), logarithmic scale.

For each temperature, the background cathode current values were taken. Next, the dependence of the background current *I*_background_ on the inverse temperature (1/*T*) was built (Figure 12). The angular coefficient of the obtained straight line also has the meaning of the activation energy of triiodide ions, and the calculation results are also listed in Table 2 to match.

The angular coefficients found by various methods are presented below in Table 2.

As it can be seen from Figure 12, the angular coefficient *k*_Ibackground_ = −1850 ± 70 coincides within the error with the activation energy for $\omega_{D_{1}}$ and $\omega_{D_{2}}$. Thus, the activation energies obtained by fundamentally different methods coincide with each other. However, the angular coefficient for the diffusion frequency $\omega_{D_{3}}$, associated with the size of the electrode, differs from the values found for other characteristic frequencies and background currents, which may indicate a slightly different nature of $\omega_{D_{3}}$ and possible cumulative effect on this typical frequency from several physical mechanisms depending on temperature. Also, this may be due to the use of a linear approximation method. Nonlinear models can much better describe the obtained experimental curves in some ways. However, for this, it is necessary to update our existing theoretical model and these are good prerequisites for further research.

## 4. Conclusions

This work presents samples of MET-based accelerometers capable of operating under conditions of significantly elevated temperatures and pressures. As a result of the research, the transfer characteristics of the MET-based sensor in an extended temperature range (25–125 °C) with an external pressure of 10 atm were recorded for the first time. Based on the experimental data, a new theoretical model was created, which allowed us to describe the MET sensor transfer function analytically in an extended temperature range at pressures up to 10 atm. A physical model was tested with several alternative techniques.

Further work on the task involved the creation of MET samples with power electromagnetic feedback and a thermal compensating filter circuit in electronics, as well as confirmation of the performance of the final product at temperatures of up to 180 °C. These products can be widely used as sensitive geophysical sensors and hydrophones for the needs of research in aggressive conditions of elevated temperatures and pressures.

## Figures and Tables

**Table 1:** Approximation parameters of $A_{0}$, $\omega_{0}$, d, $\omega_{D_{1}}$,$\;\omega_{D_{2}}$, $\omega_{D_{3}}$, for each of the temperatures, pressure 10 atm.

T, °C	$\omega_{D_{1}},\;\mathrm{Hz}$	$\;\omega_{D_{2}},\;\mathrm{Hz}$	$\omega_{D_{3}},\;\mathrm{Hz}$	$\omega_{0},\;\mathrm{Hz}$	d, s^−1^	$A_{0},\;\mathrm{Reading}\;\mathrm{from}\;\mathrm{AD}\;\mathrm{Converter}/m/s^{2}$
*25*	0.11	0.72	19.13	357.96	0.76	29,493
*35*	0.13	0.79	21.32	362.92	0.74	30,980
*45*	0.16	0.88	24.25	355.26	0.68	29,977
*55*	0.20	1.03	29.30	347.73	0.61	28,958
*65*	0.20	1.28	34.53	345.20	0.55	29,165
*75*	0.22	1.51	39.85	338.40	0.51	28,840
*85*	0.22	1.92	42.09	331.96	0.47	28,446
*95*	0.27	2.17	47.45	325.42	0.45	27,343
*105*	0.35	2.51	46.44	312.83	0.45	25,427
*115*	0.42	2.75	48.02	292.16	0.40	24,608
*125*	0.59	2.80	50.01	297.98	0.38	24,341

**Table 2:** The values of the angular coefficients for each of the approximation parameters $\omega_{D_{1}}$,$\;\omega_{D_{2}}$, $\omega_{D_{3},}$ and $I_{background}$.

	$\mathrm{Activation}\mathrm{Energy}[E_{a}/k\;({}^{0}К)]$	*Error*
$\omega_{D_{1}}$	−1730	163
$\;\omega_{D_{2}}$	−1820	79
$\;\omega_{D_{3}}$	−1230	88
$\;I_{background}$	−1850	70
